# Do MCAT scores predict USMLE scores? An analysis on 5 years of medical student data

**DOI:** 10.3402/meo.v21.31795

**Published:** 2016-09-30

**Authors:** Jacqueline L. Gauer, Josephine M. Wolff, J. Brooks Jackson

**Affiliations:** Medical School, University of Minnesota, Minneapolis, MN, USA

**Keywords:** MCAT, USMLE, admissions, multiple linear regression, predictive value, chi-square

## Abstract

**Introduction:**

The purpose of this study was to determine the associations and predictive values of Medical College Admission Test (MCAT) component and composite scores prior to 2015 with U.S. Medical Licensure Exam (USMLE) Step 1 and Step 2 Clinical Knowledge (CK) scores, with a focus on whether students scoring low on the MCAT were particularly likely to continue to score low on the USMLE exams.

**Method:**

Multiple linear regression, correlation, and chi-square analyses were performed to determine the relationship between MCAT component and composite scores and USMLE Step 1 and Step 2 CK scores from five graduating classes (2011–2015) at the University of Minnesota Medical School (*N*=1,065).

**Results:**

The multiple linear regression analyses were both significant (*p*<0.001). The three MCAT component scores together explained 17.7% of the variance in Step 1 scores (*p<*0.001) and 12.0% of the variance in Step 2 CK scores (*p*<0.001). In the chi-square analyses, significant, albeit weak associations were observed between almost all MCAT component scores and USMLE scores (Cramer's V ranged from 0.05 to 0.24).

**Discussion:**

Each of the MCAT component scores was significantly associated with USMLE Step 1 and Step 2 CK scores, although the effect size was small. Being in the top or bottom scoring range of the MCAT exam was predictive of being in the top or bottom scoring range of the USMLE exams, although the strengths of the associations were weak to moderate. These results indicate that MCAT scores are predictive of student performance on the USMLE exams, but, given the small effect sizes, should be considered as part of the holistic view of the student.

Medical school admissions committees have long debated how best to select students for their programs. While the Association of American Medical Colleges is advocating for a holistic approach to admissions decisions (1), challenges remain when it comes to assessing personal qualities for admissions decisions (2), including difficulties selecting and defining the qualities to select for, cost-effectiveness in measurement, and the inherent subjectivity of evaluating such qualities. Due to its reputation as a relatively objective and standardized measure of preparation for medical school, the Medical College Admission Test (MCAT), a multi-component standardized exam, remains one of the most influential components of an application to medical school. Research supports the continued use of the MCAT in admissions decisions, having found the MCAT to be more consistently valuable for predicting measures of medical school success than other frequently-considered factors such as grade point average (GPA) (3). The MCAT can also serve as a useful leveling factor when evaluating applicants from undergraduate institutions of differing selectivity (4). With the release of a new version of the MCAT in 2015, it has become increasingly important to understand the predictive value of the MCAT on student success.

One outcome that is frequently used to measure medical student success is the student's score on the U.S. Medical Licensure Exam (USMLE). The USMLE is a three-step examination sponsored by the Federation of State Medical Boards and the National Board of Medical Examiners^®^ required for physician licensure for all physicians, regardless of training location, to practice in the United States. Typically, for medical students receiving training at LCME-accredited institutions in the US and Canada, students take Step 1 of the USMLE at the end of the second year of medical school, Step 2 Clinical Knowledge (CK) and Clinical Skills (CS) in the fourth year of medical school, and Step 3 after medical school. Together, Step 1 and Step 2 CK assess a physician's ability to apply knowledge and concepts to provide safe and effective patient care. Step 1 assesses whether medical students understand and are able to apply important concepts of basic science to medical practice, with special emphasis on principles of underlying modes of therapy, health, and disease. Step 2 CK further assesses health promotion and disease prevention and incorporates the principles of clinical sciences and basic patient-centered skills for safe practices of medicine (5).

A substantial body of literature supports the predictive value of MCAT scores on USMLE scores. Ogunyemi and Taylor-Harris (6) found a significant positive correlation between MCAT scores and USMLE Step 2 scores (*r*=0.524, *p*=0.000). In their study, MCAT scores correlated more strongly with Step 2 scores than did undergraduate GPA (*r*=0.287, *p*=0.000) and science GPA (*r*=0.255, *p*=0.002). Researchers at the Jefferson Medical College found that MCAT scores were moderately correlated with both medical school performance and USMLE scores in a meta-analysis of the literature (7), concluding that the MCAT composite score had strong predictive validity for USMLE Step 1 and medium predictive validity for USMLE Step 2 (8). Preliminary analyses of the MCAT version released in 1991 (the version under analysis in this study) found that the MCAT exam accounted for a significant portion of the variance in USMLE Step 1 scores, and that including undergraduate information, such as GPA and undergraduate institution selectivity, added little to the model (9). The same analysis found that, of the MCAT components, the Biological Science (BS) and Physical Science (PS) components were most predictive, and Verbal Reasoning (VR) and Writing Sample (WS) component scores contributed little to predictive accuracy. Basco et al. (10) also found that USMLE Step 1 scores were differentially correlated with individual components of the MCAT (*r*=0.491 for PS, *r*=0.553 for BS, and *r*=0.397 for VR).

Some research, on the other hand, indicates that the predictive value of MCAT scores on USMLE scores may not be consistent and depends on several factors. One study found evidence that, while the predictive value of the MCAT on USMLE Step 1 and Step 3 has remained stable, the MCAT's predictive value for USMLE Step 2 decreased with each new MCAT version over the course of 36 matriculating classes at the Jefferson Medical College (7). Furthermore, the predictive validity of the MCAT has been shown to be different as a function of different undergraduate institutions (11), the number of times students have taken the exam (12), and the ethnicity of the exam-takers (13). Although the literature supporting moderate predictive value for the MCAT on medical school performance is robust, these findings indicate that the predictive validity of the MCAT remains an important issue to continue to explore, especially considering that each medical school operates in a different context.

Although most of the current research on MCAT and USMLE scores considers the full range of participants’ scores, admissions committees are most concerned with the students at the extreme ends of the scoring ranges and are less concerned with middle ranges. For students scoring in the middle range of those generally accepted by the institution, the MCAT may not be an important factor for admission. However, admissions committees faced with a candidate who has a low MCAT score but an otherwise strong application need to know whether that student's low MCAT score should be sufficient to preclude that candidate from admission to the institution. They wonder whether other impressive components of the application might be able to compensate for a low MCAT score in indicating the candidate's potential to succeed in medical school. However, since success on the USMLE is necessary for licensure, low USMLE scores can outweigh other measurements of success, such as course grades or even clinical performance, if they preclude the student from licensure or from a successful residency match. Therefore, it is important to determine whether a student who scores in the bottom range of MCAT scores is particularly likely to also score in the bottom range of USMLE scores.

This study expands upon the previous research by exploring the predictive value of being a particularly-high or particularly-low scorer using a chi-square model. Since the participants in this study were admitted medical students, we can assume that the students with low MCAT scores did indeed have other impressive application components that lead to their admission, thus allowing us to explore whether those factors allowed for a rebound in performance on the USMLE exams, or whether those students continued to score poorly. For comparison, we also included the parallel analysis of whether students scoring high on the MCAT continue to score high on the USMLE exams. We cannot, however, make the parallel assumption that those students with high MCAT scores had other weaker application components that were compensated for by the MCAT scores in the admissions decision, since their admittance may simply have been a reflection of all-around excellence.

The overall objective of this study was to determine the associations and predictive values of component and composite MCAT scores with USMLE Step 1 and Step 2 CK scores. We hypothesized that the individual MCAT components would be predictive of USMLE scores in a regression equation, but that different components would have different predictive values for USMLE scores. Furthermore, we hypothesized that being in the top or bottom range of MCAT scores on each component would be predictive of being in the top or bottom range of USMLE scores.

## Methods

### Institutional approval

Ethical approval for this research was granted by the Institutional Review Board at the University of Minnesota.

### Participants

The participants for this study included all students pursuing MD (*N*=1,060) and MD/PhD (*N*=5) degrees at the University of Minnesota who both matriculated between 2007 and 2011 and graduated between 2011 and 2015 (Total *N*=1,065). Of the included students, 545 (51.2%) were male, and 520 (48.8%) were female. Age at matriculation ranged from 19 to 42 years (M=23.7 years, SD=2.6 years). At the University of Minnesota, medical students matriculate at either the Duluth campus or the Twin Cities campus. They complete the first 2 years of the degree (basic science courses) at their campus of matriculation, and then all students complete the second 2 years of the degree (clinical clerkships) through the Twin Cities campus. Of the students in this study, 281 (26.4%) matriculated at the Duluth campus and 784 (73.6%) matriculated at the Twin Cities campus. Four students did not take the USMLE Step 2 exam, and their data are not included in results involving scores from that exam.

### Sources of data

Academic and demographic data were collected from student records held by the Office of Medical Education in the Academic Health Center at the University of Minnesota. Data regarding participants’ MCAT composite and component scores were retrieved from the University of Minnesota's access to primary application data through the American Medical College Application Service (AMCAS). The WS MCAT component was not analyzed individually in this study, as previous research has shown it to have minimal predictive validity for USMLE scores (although it may have predictive validity for clinical performance) (8, 14, 15). For students who had multiple MCAT scores, the highest composite score was utilized, as this is the score that is given most weight by the admissions committee of the institution under analysis. Data regarding participants’ USMLE Step 1 and Step 2 CK scores were provided to the Medical School by the National Board of Medical Examiners upon students’ completion of each exam.

### Analyses

Using SPSS Statistics v.22 (IBM, Armonk, NY), we performed analyses on the collected data to explore possible predictive relationships between MCAT component and composite scores and the USMLE Step 1 and USMLE Step 2 CK exams.

We calculated Pearson product-moment correlation coefficients for MCAT composite scores with USMLE Step 1 and Step 2 CK scores.

To determine the relationships between MCAT component scores and each of the USMLE exams under examination, we completed a multiple linear regression for each USMLE exam. We performed successful checks of multicollinearity, normality, and outliers for each analysis to ensure that multiple linear regression was an appropriate model to use with these data.

We also explored the possible predictive value of MCAT component and composite scores on USMLE Step 1 and Step 2 CK scores using chi-square tests of association. Since admissions committees tend to give more weight to particularly high or low scores in admissions decisions, we were interested in seeing if being a particularly high- or low-scorer on the MCAT was predictive of also being a particularly high- or low-scorer on the USMLE exams. In order to establish high- and low-scoring groups, we determined the top and bottom scorers on each exam and on each component. Since cutoff scores were not available at precisely the same point for each exam, due to the limited range of scores, we determined the closest cutoffs to the top 20% and bottom 20% of each. We will refer to the groups created by these cutoffs as the Top Group and Bottom Group scorers of each exam and MCAT component. The exact percentage of scorers in each Group can be seen in Table 1. We created two-by-two crosstab tables to determine chi-square and *p*-values for each of the Top Groups of MCAT scores with each of the Top Group of USMLE scores, and for each of the Bottom Group of MCAT scorers with each of the Bottom Group USMLE scorers. We calculated Cramer's V values to determine the strength of each relationship.

**Table 1:** Cutoff scores used to determine top and bottom scoring groups of students pursuing MD degrees at the University of Minnesota who both matriculated between 2007 and 2011 and graduated between 2011 and 2015 (*N*=1,065)

Exam	MCAT Composite	MCAT Biological Science	MCAT Physical Science	MCAT Verbal Reasoning	USMLE Step 1	USMLE Step 2 CK
Top score Cutoff	≥35 (17.7%)	≥13 (13.3%)	≥12 (26.5%)	≥12 (15.5%)	≥246 (17.6%)	≥254 (18.3%)
Bottom score Cutoff	≤28 (17.8%)	≤9 (15.9%)	≤8 (12.4%)	≤8 (14.2%)	≤207 (17.9%)	≤222 (18.1%)

## Results

### Correlations

We computed Pearson product-moment correlation coefficients to assess the general relationship between MCAT composite scores and USMLE exam scores. We found positive correlations for both Step 1 (*r*=0.39, *p*<0.001) and Step 2 CK (*r*=0.31, *p*<0.001). These results indicate significant moderately positive relationships between MCAT composite scores and USMLE exam scores.

### Multiple linear regressions

We conducted multiple linear regression analyses to determine the relationships between MCAT component scores and scores on the USMLE Step 1 and Step 2 CK exams. We calculated a multiple linear regression to predict USMLE Step 1 scores based on MCAT BS, PS, and VR component scores. We found that the BS (β=0.277, *p*<0.001), PS (β=0.199, *p<*0.001), and VR (β=0.062, *p*=0.031) components were all significant predictors. The three predictor model was able to account for 17.7% of the variance in Step 1 score, *F*(3, 1,061)=75.862, *p*<0.001, *R*_2_=0.177. Students’ predicted USMLE Step 1 score is equal to 157.155+3.548(BS)+2.215(PS)+0.748(VR), where BS is the student's score on the MCAT BS component, PS is the student's score on the MCAT PS component, and VR is the student's score on the MCAT VR component. USMLE Step 1 score increased 3.548 points for each point on the MCAT BS component, 2.215 points for each point on the MCAT PS component, and 0.748 points for each point on the MCAT VR component.

We also calculated a multiple linear regression to predict USMLE Step 2 CK scores based on MCAT BS, PS, and VR component scores. We again found that the BS (β=0.253, *p*<0.001), PS (β=0.085, *p=*0.007), and VR (β=0.118, *p*<0.001) components were all significant predictors. The three predictor model was able to account for 12.0% of the variance in Step 2 score, *F*(3, 1,057)=48.032, *p*<0.001, *R*2=0.120. Students’ predicted USMLE Step 2 CK score is equal to 186.324+2.819(BS)+0.822(PS)+1.237(VR), where BS is the student's score on the MCAT BS component, PS is the student's score on the MCAT PS component, and VR is the student's score on the MCAT VR component. USMLE Step 2 CK score increased 2.819 points for each point on the MCAT BS component, 0.822 points for each point on the MCAT PS component, and 1.237 points for each point on the MCAT VR component.

### Chi-square tests of association

We conducted chi-square tests of association to determine the predictive values of MCAT composite and component scores on the USMLE Step 1 and Step 2 CK exams. The strengths of the associations between being in the Top or Bottom Group on each MCAT component and being in the Top or Bottom Group on USMLE Step 1 and Step 2 CK are shown in Table 2.

**Table 2:** Results of chi-square tests of association between top/bottom scorers in each MCAT component and top/bottom scorers of USMLE Step 1 and Step 2 CK, for students pursuing MD degrees at the University of Minnesota who both matriculated between 2007 and 2011 and graduated between 2011 and 2015 (*N*=1,065)

MCAT score	USMLE exam	Score category	Chi-square	*p* (2-sided)	Cramer's V
Composite	Step 1	High scorers	38.87	<0.001	0.19
		Low scorers	62.60	<0.001	0.24
	Step 2 CK	High scorers	21.72	<0.001	0.14
		Low scorers	54.11	<0.001	0.23
Biological sciences	Step 1	High scorers	40.55	<0.001	0.20
		Low scorers	50.27	<0.001	0.22
	Step 2 CK	High scorers	19.86	<0.001	0.14
		Low scorers	46.21	<0.001	0.21
Physical sciences	Step 1	High scorers	29.88	<0.001	0.17
		Low scorers	24.87	<0.001	0.15
	Step 2 CK	High scorers	5.76	0.02	0.07
		Low scorers	21.51	<0.001	0.14
Verbal reasoning	Step 1	High scorers	2.33	0.13	0.05
		Low scorers	18.31	<0.001	0.13
	Step 2 CK	High scorers	10.30	0.001	0.10
		Low scorers	19.32	<0.001	0.14

As can be seen in Table 2, *p*-values were at or below 0.001 for all except three comparisons. The associations were significant at the *p*<0.05 level for all except one comparison. We tested the strengths of the associations with Cramer's V. Cramer's V is a test of the strength of an association, similar to a correlation. Possible values of Cramer's V range from 0 to 1, with values below 0.1 generally considered to be indicative of a very weak association, 0.1–0.2 a weak association, 0.2–0.3 a moderate association, and 0.3 and above a strong association. Cramer's V values for these comparisons ranged from 0.05 to 0.24, indicating very weak to moderate associations.

For the sake of comparison, we also investigated the percent of the Top Group of scorers on the MCAT who also scored in the top half of USMLE Step 1 and Step 2, as well as those who had a composite score of 40 or above on the MCAT (*N*=9). For Step 1, the percent of the Top Group of MCAT scorers who also scored in the top half of Step 1 scores was 70.9%. The percent of those who scored 40 or above on the MCAT who also scored in the Top Group of Step 1 scorers was 88.9%. The percent of those who scored 40 or above on the MCAT who also scored in the top half of Step 1 scores was 100%. For Step 2, the percent of Top Group MCAT scorers who also scored in the top half of Step 2 CK scores was 66.1%. The percent of those who scored 40 or above on the MCAT who also scored in the Top Group of Step 2 CK scorers was 66.7%. The percent of those who scored 40 or above on the MCAT who also scored in the top half of Step 2 CK scores was 100%.

## Discussion

Overall, the results of this study are consistent with previous literature that shows that MCAT scores are predictive of performance on the USMLE Step 1 and USMLE Step 2 CK exams. The results of the analyses were significant overall, but the effects were small to moderate, indicating that MCAT scores should not be the sole factor considered in admissions decisions. Indeed, arguments have been made that test scores are not reflective of eventual physician performance, and that noncognitive factors, such as altruism and a sense of duty, should also be considered as selection criteria and featured prominently in physician training (1, 16). There is also the possibility that the correlation between MCAT and USMLE scores is more related to simple test-taking ability than to potential success as a physician. Our findings could therefore be taken to indicate that, instead of precluding a candidate from admission, a low MCAT score could instead be used as an indicator to flag incoming students for increased training in specific USMLE-related topics and/or test-taking strategies.

We found that MCAT component scores, taken together in a multiple linear regression model, were predictive of USMLE Step 1 and Step 2 CK scores. The three components together accounted for 17.7% of the variance in Step 1 scores and 12.0% of the variance of Step 2 CK scores. Therefore, while the models were both significant (*p*<0.001), the effect sizes were relatively small. The three MCAT components differentially predicted scores on each USMLE exam. For USMLE Step 1, the MCAT BS component was most predictive, followed by the MCAT PS component, and finally the MCAT VR component. The MCAT BS component was also the most predictive component for USMLE Step 2 CK, but the MCAT VR component was more predictive than the MCAT PS component.

We found MCAT component and composite scores to have overall predictive value for scoring in the top or bottom group of USMLE Step 1 and Step 2 CK scores. Chi-square tests of association found that being in the Top Group of scorers on the MCAT composite or MCAT components was significantly associated with being in the Top Group of scorers on USMLE Step 1 and USMLE Step 2 CK for all comparisons except between the VR MCAT component and Step 1. There were even stronger associations between being in the Bottom Group of scorers on each exam. Overall, it appears from these data that composite scores had the strongest association with Step 1 and Step 2 CK scores, followed by the BS component, the PS component, and the VR component, in that order. Furthermore, the low scoring group comparisons showed higher overall values of Cramer's V than their counterpart high scoring group comparisons, indicating that being a low scorer on the MCAT was more predictive of being a low scorer on Step 1 and Step 2 CK than being a high scorer on the MCAT is predictive of being a high scorer on Step 1 and Step 2 CK. This finding indicates that it may be worthwhile for admissions committees to give particular scrutiny to candidates with low MCAT scores. It is important to note that, although most of the comparisons were significant, the Cramer's V values indicated only weak to moderate associations, indicating that many other factors come into play in determining Step 1 and Step 2 CK scores.

The results of the Pearson product-moment correlation coefficients between MCAT composite scores and Step 1 and Step 2 CK scores showed that MCAT composite scores are significantly correlated with both Step 1 and Step 2 CK scores, but that the strengths of those correlations are fairly weak. This finding provides further evidence that level of performance on the MCAT is associated with level of performance on Step 1 and Step 2 CK, but that the actual size of the effect is fairly small.

Our findings that MCAT scores are associated with USMLE scores reflect the results from a study by Julian (17), which found that MCAT scores were strong predictors for all three USMLE Step exams. Julian also included undergraduate GPAs in her regression analyses and found that while USMLE scores were best predicted by undergraduate GPA and MCAT scores combined, the combination was not a much better predictor than MCAT scores alone, and that MCAT scores alone were better predictors than undergraduate GPA alone. Our findings expand on Julian's results by exploring the relationships of individual MCAT components to USMLE scores, and by determining that scoring low seems to be more predictive of continuing to score low than scoring high is predictive of continuing to score high. This finding could help medical schools decide who to target for programs that offer additional support or USMLE preparation.

As with any study of this type, we must be careful of the claims we make from the findings. The nature of the observational data collection method is that we cannot draw causal conclusions about our findings, only make claims of associations. Across all the analyses included in this study, it is important to note that although many of the results were highly significant, we must also consider the practical implications of, for example, a 0.822 point increase on USMLE Step 2 CK for each MCAT PS point. Some of these effects may be highly significant, due to the large sample size included in the study, but the actual difference may be negligible. It continues to remain important to use these findings in the context of holistic review. Furthermore, this study used only the highest reported MCAT score for each student, as that is the score considered by the admissions committee at the institution under study. However, previous research has shown that taking the MCAT multiple times does affect the predictive value of MCAT scores (12). Individual institutions should consider this factor when determining how to apply these findings.

As of April 2015, the Association of American Medical Colleges launched an updated version of the MCAT exam, which includes the following sections: Biological and Biochemical Foundations of Living Systems; Chemical and Physical Foundations of Biological Systems; Psychological, Social, and Biological Foundations of Behavior; and Critical Thinking and Reasoning Skills (18). In addition to the different sections of the exam, most notably the addition of the new behavioral and social sciences component, there was also a significant increase in the number of questions and total content time. The release of the new version of the MCAT has implications for the applicability of the current research. However, we believe that our data provide a foundational background on the old MCAT design that will facilitate analysis of the new design, especially in comparison to the old design.

In conclusion, this study provides an analysis, using a large dataset of over 1,000 medical students covering 5 years of medical school education, of the predictive value of MCAT scores on USMLE Step 1 and Step 2 CK scores. The results of the analyses indicate that MCAT component and composite scores do have significant predictive value for the USMLE Step 1 and Step 2 CK exams, with the caveat that the effect sizes found were small and may or may not be practically significant. These findings replicate some of the previous research confirming the predictive value of the MCAT on USMLE scores and have implications for the future use of the MCAT in admissions decisions, particularly when it comes to students with low MCAT scores. This study also provides a foundational background on the old MCAT design which may help facilitate analysis of the new design.
